# Full disclosure: Genome assembly is still hard

**DOI:** 10.1371/journal.pbio.2005894

**Published:** 2018-04-24

**Authors:** Stephen Richards

**Affiliations:** Human Genome Sequencing Center, Baylor College of Medicine, Houston, Texas, United States of America

## Abstract

Two recent papers highlight the fascinating comparative genomics of anhydrobiosis, the ability to withstand complete desiccation, in bdelloid rotifers and tardigrades. However, both groups had to openly deal with the significant difficulties of generating and interpreting short-read draft assemblies—especially challenging in microscopic species with high sequence polymorphism. These exemplars demonstrate the need to go beyond single draft-quality reference genomes to high-quality multiple species comparative genomics if we are to fully capture the value of genomics.

## Introduction

I recently read Richard Feynman’s commencement address, “Cargo Cult Science” [1], in which the famous physicist reminds us that scientific integrity demands “a kind of utter honesty—a kind of leaning over backwards” and that “if you’re doing an experiment, you should report everything that you think might make it invalid—not only what you think is right about it.” While we aspire to this ideal as a matter of course, certain data types can grow familiar, and one can assume a general quality until stumbling upon its limits. Lately, this seems especially so in analyzing short-read draft reference assemblies, where many researchers are making do, trying to generate insights from single taxa with fragmented sequences.

**Fig 1:**
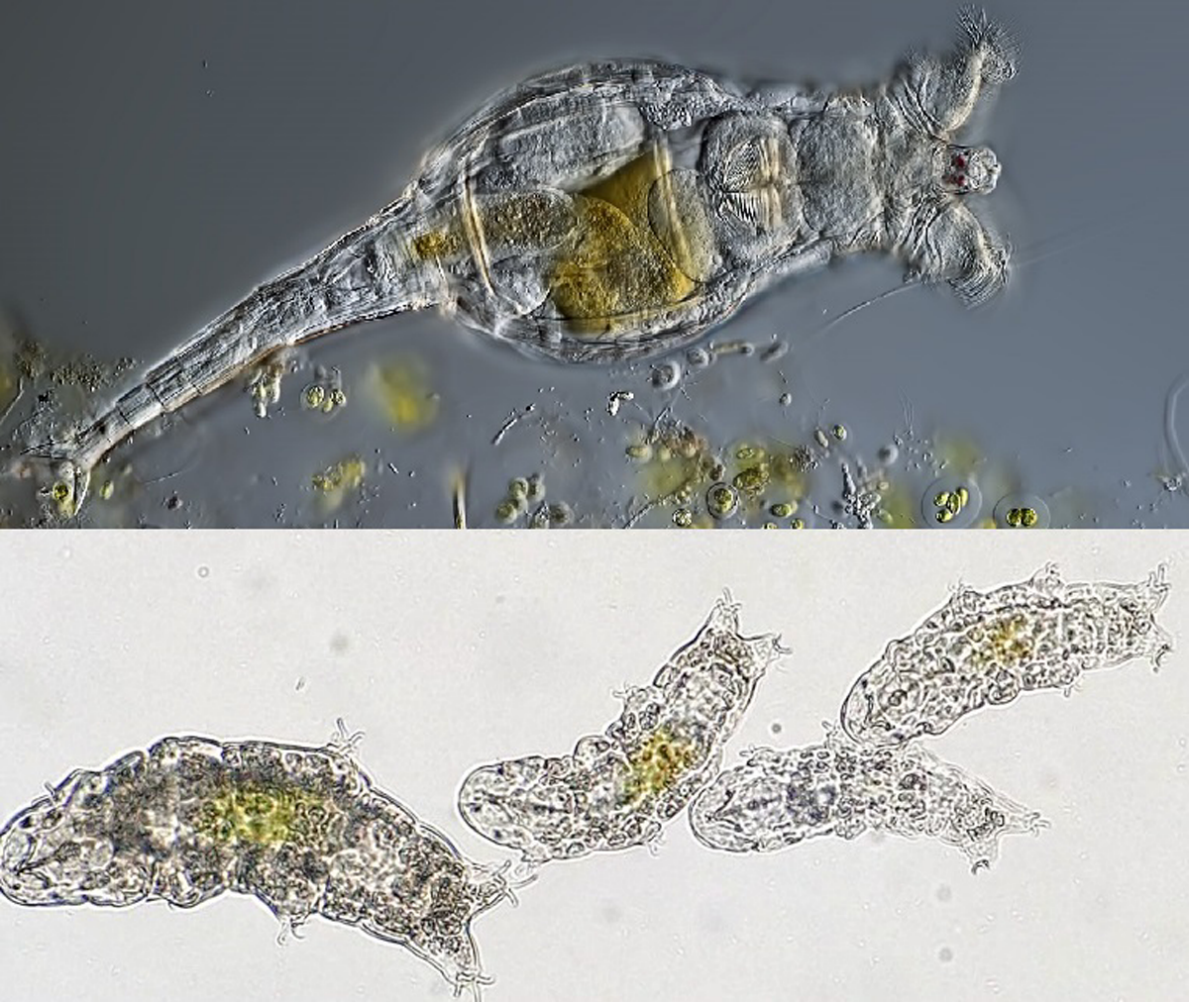
Photomicrographs of 2 of the animals discussed here. The bdelloid rotifer *Rotaria macrura* (top) and the tardigrade *Hypsibius dujardini* (bottom). Image credits: Michael Plewka and Kazuharu Arakawa.

Nowell et al. [2] (this issue of *PLOS Biology*) and Yoshida et al. [3] both utilize additional comparative genomes with upgraded quality to significantly advance our understanding of the bdelloid rotifers and tardigrades, respectively (Fig 1). These fascinating extremophiles withstand X- and gamma-ray exposure, and most interestingly, some survive complete desiccation (anhydrobiosis) in adaptation to living in water film environments that literally evaporate away. They share microscopic body sizes, which restricts DNA yield from single individuals, and high polymorphism—both characters complicating short-read sequence generation and genome reference assembly. Additionally, the tardigrade phylum resists easy taxonomic placement, and the bdelloid rotifers display a curiously long-lived asexuality—they lack males and replicate by mitotic parthenogenesis, and yet, the lineage has survived for tens of millions of years.

### Bdelloid comparative genomics

Nowell et al.’s bdelloid work epitomizes Feynman’s description of scientific integrity as they successfully grapple with the difficulties and uncertainties of short-read assembly. The first pioneering draft assembly of the rotifer *Adineta vaga* [4] suggested that conventional meiosis was incompatible with the scrambled genome structure preventing segregation of haploid sets, found high levels of horizontal gene transfer (HGT) from nonmetazoans, and hypothesized high levels of gene conversion after double-stranded break repair of desiccation-damaged DNA. The current work tests this hypothesis by comparing species that can and cannot survive desiccation. This clearly requires some faith in the underlying genomes, and utterly true to Feynman’s admonitions of honesty to Caltech’s graduating class of 1974, they have described to the best of their ability all of the issues with their genome reference assemblies upon which their conclusions are based. Before going further, I must impress on you that these draft assemblies are quite good. This author (SR) has produced worse on difficult species. Here, a combination of long and short reads generated contig N50s ranging from 50–270 kb for most of the assemblies (N50 is a sequence-length assembly statistic in which 50% of the assembly is contained in sequences greater than or equal to this “N50” length.) Also, bdelloid rotifers exemplify the possible problems with short-read assembly. In addition to the microscopic size and high polymorphism described above, these species are tetraploid, thus increasing the likelihood of failing to collapse polymorphic alleles into a single sequence for the reference genome.

The authors addressed how every assembly deficiency may affect their conclusions. To address the possibility that polymorphic sequences from 4 haplotypes may or may not assemble into a single reference, they generated 2 assemblies. The first attempted to maximally collapse the haplotypes to a single reference using assembly tools designed for that task—Platanus [5] and Redundans [6]. At the other extreme, they minimized assembly collapse to create maximum haplotype assemblies. This generated a range of estimates for genome size, gene number, and repetitive content, and annoyingly, 2 assemblies to choose from when studying the organism. This is disconcerting! We are used to definitive numbers (likely conveying overconfidence) and instead feel like the proverbial man with 2 watches who doesn’t know the time. But the authors have fully conveyed the limit of their confidence in their conclusions, and any uncertainty left with the reader is precisely defined. They find that bdelloids able to survive desiccation have twice as many genes and show much higher levels of homologous divergence than species unable to do so, a result that is contrary to previous hypotheses. They confirm high levels (approximately 10%) of HGT from nonmetazoans in multiple species.

### Tardigrade genomics

In the tardigrade analysis, Yoshida et al. compared the genomes of 2 tardigrades: *Ramazzottius varieornatus*, which is able to survive rapid desiccation, and *Hypsibius dujardini*, which requires preconditioning to do so [3]. The first published *H*. *dujardini* assembly [7] claimed (perhaps now infamously) that one-sixth of the genome assembly was due to HGT from bacteria to this eukaryote. This result was immediately challenged by others in the field [8,9], and careful filtering and cautious reassembly of the short-read data reduced the suspected amount of HGT to approximately 1%–2% of the assembled genome. Normal HGT levels were confirmed by Yoshida et al., who required a hybrid assembly based on short and long reads, DNA from a culture for long reads, and single individual genome amplification for short reads, to resolve high heterozygosity. The resulting assembly had contig N50s of 342 kb for *H*. *dujardini* reference 3.0 compared to 15.2 kb for the original [7], with minimal heterozygous regions. Note that higher-quality assemblies reduce the possibility of such analytical missteps. Yoshida et al. carefully compared references and transcriptomes in a tour de force to identify mechanisms underlying tardigrade anhydrobiosis. Highlights include extensive gene loss in the mammalian target of rapamycin (mTOR) pathways, up-regulation of reactive oxygen protection loci, and expansion of gene families such as chaperones and DNA repair endonucleases. Such detailed analysis simply requires additional work beyond the first genome analysis.

The authors also addressed the still controversial taxonomic placement of tardigrades, which are either sister to arthropods, based more on morphological data, or to nematodes, based more on molecular data. Perhaps surprisingly, at genome scale they find more molecular support for the tardigrade-nematode sister relationship (HOX gene loss patterns matching the nematodes being particularly convincing) but that rare genomic changes supported tardigrade-arthropods. Fundamentally, our simple conception of the phylogenetic tree may have to be updated as different datasets support different groupings. This work generated a detailed accounting of which tardigrade proteins support which placement. Similar phylogenetic data incongruence is appearing all over the tree of life (see [10] for a review of the multiple ways this can happen).

### Problems with short-read draft assemblies

As a researcher who has generated many short-read draft assemblies, some with very low contiguity, in the spirit of Feynman’s utter honesty, I must come clean. Usually, and especially in the case of species with no close relatives, we do not know the correct answer as we construct genome assemblies blindly from short sequences. Divergent haplotypes overlapping with repeats lead to fragmented short-read assemblies in which short contigs adversely affect gene annotation. Those studying telomeres, centromeres, and more diverse heterochromatic regions are not served by these draft assemblies. If the assembly is too fragmented, validation techniques such as optical and BioNano genome maps will not have long enough assembly fragments to assess and in any case will significantly increase costs. Although gene models are generated using RNA sequencing (RNA-Seq) and protein comparison data, they are models—i.e., hypotheses with differing levels of supporting data. The number of gene models depends on both the quality of the assembly (fragmented genomes lead to fragmented and perhaps double-counted gene models), the quality of the RNA-Seq data (which can join gene models and confuse alternate transcripts), and the parameters and definitions of a gene encoded in the software (which, tuned for the average gene, cannot perform well on all). Genes with low expression (perhaps restricted to a small tissue), rapid divergence, or unique genes will have less alignment evidence from both transcripts and proteins and may not be modelled in automated pipelines. It is routine for automated gene sets to miss perhaps 5% of genes in draft genomes, and manual curation rarely assesses more than a favored 10% of gene models. I do not wish to overplay these problems; the era of the short-read draft assembly has transformed biology. Thanks to the conscientiousness of the researchers creating assembly tools, contig and scaffold sequence quality is very high, and the vast majority of errors present as gaps in the sequence. In vertebrates (large, charismatic species with low sequence polymorphism), high-quality draft genomics have been informative in the study of birds [11] and marine mammals [12], and in cheetahs, they have aided conservation understanding [13], to list a few recent highlights.

New long-read sequencing technologies, polymorphism-aware assemblers, and chromatin mapping Hi-C sequencing are enabling previous draft references and conclusions based upon them to be revisited. Often the first genome sequence in a clade may generate a high-profile publication, but because of the techniques available at that time, it may not be of the highest quality. In “The importance of being second” [14], the *PLOS Biology* staff editors remind us of the importance of replication and extension of research results. Analogously, it is critical that significantly improved references with additional species and updated comparative analyses be publishable. Indeed, these references will be the foundation of research for the biology community, so investments will be amortized for years to come.

It is timely to address these issues. The Earth BioGenome Project [15] is a new initiative to sequence all species on earth. To generate high-quality biological genome references at this scale, the problems of producing genome references from small amounts of polymorphic DNA must be resolved. Recent progress in this area includes extremely long reads [16], partition libraries requiring extremely little DNA [17], the use of parental sequences to reduce haploidy in long-read reference assemblies [18], and ongoing efforts by long-read companies to reduce the input DNA amounts required. In the meantime, however, careful interpretation of short-read draft genome references with full disclosure will be required.

## Abbreviations

HGT: horizontal gene transfer
mTOR: mammalian target of rapamycin
RNA-Seq: RNA sequencing
